# JC virus granule cell neuronopathy onset two months after chemotherapy for low-grade lymphoma

**DOI:** 10.1186/s40673-017-0066-6

**Published:** 2017-06-23

**Authors:** Kathryn B. Holroyd, Elias S. Sotirchos, Scott R. DeBoer, Kelly A. Mills, Scott D. Newsome

**Affiliations:** 1 Johns Hopkins University School of Medicine, Baltimore, MD USA; 2 Department of Neurology, Johns Hopkins University School of Medicine, Baltimore, MD USA; 3 Division of Neuroimmunology and Neurological Infections, Johns Hopkins Hospital, 600 North Wolfe St., Pathology 627, Baltimore, MD 21287 USA

**Keywords:** Cerebellar degeneration, Ataxia, Granule cell neuronopathy, Progressive multifocal leukoencephalopathy, Rituximab, Lymphoma

## Abstract

**Background:**

Granule cell neuronopathy (GCN) is a rare disease caused by the JC virus, leading to degeneration of cerebellar granule cell neurons. Primarily described in patients with AIDS, it has also been diagnosed in patients with lymphoproliferative diseases and after long-term treatment with immune-suppressing medications such as natalizumab.

**Case presentation:**

A 69 year old woman presented with progressive ataxia which began 2 months after initiation of treatment for follicular low-grade B cell lymphoma with rituximab/bendamustine, and progressed for 2 years prior to admission. Extensive prior evaluation included MRI that showed atrophy of the cerebellum but normal CSF analysis and serum studies. Neurologic exam on admission was notable for severe appendicular ataxia and fatigable end-gaze direction-changing horizontal nystagmus. FDG-PET/CT scan was unremarkable and repeat lumbar puncture revealed 2 WBCs/mm^3^, 148 RBCs/mm^3^, glucose 70 mg/dL, protein 37.7 mg/dL and negative flow cytometry/cytopathology. Standard CSF JC virus PCR testing was negative, but ultrasensitive TaqMan real-time JC virus PCR testing was positive, consistent with JC virus-related GCN.

**Conclusions:**

Because of the diagnostic challenges in identifying GCN, a high threshold of suspicion should be maintained in patients with an immune-suppressing condition such as lymphoma or on immune-suppressing agents such as rituximab, even shortly after initiation of therapy.

## Background

Granule cell neuronopathy (GCN) is a rare disease caused by the JC virus that is characterized by lytic infection of cerebellar granule cell neurons. While up to 51% of patients with progressive multifocal leukoencephalopathy (PML) show infection of granule cells by JC virus on autopsy, GCN is thought to be a distinct entity caused by mutation in the VP1 gene of the JC virus leading to a shift in viral tropism from glial cells to cerebellar granule cells [1]. GCN typically presents with chronic progressive gait ataxia, dysarthria, and incoordination, and can present in isolation or together with PML. MRI findings are characterized by cerebellar atrophy with or without cerebellar white matter lesions [2].

In the pre-AIDS era, nearly two thirds of PML cases were attributed to lymphoproliferative diseases, mainly B-cell disorders. PML became increasingly recognized in patients with the development of AIDS, and GCN as a distinct entity was characterized in this population, though it has also been described after treatment with natalizumab, rituximab (after 18 years of treatment), and in one patient with sarcoid [3, 4]. Previously, GCN has only been reported after long-term treatment with immune-suppressing agents. In the case presented here, we describe GCN that manifested just 2 months after initiation of rituximab/bendamustine for lymphoma.

## Case presentation

A 69 year old woman was diagnosed with CD20+ follicular grade 2 non-Hodgkin’s lymphoma, stage 3a in 2014. She underwent 6 cycles of rituximab 650 mg/bendamustine 160 mg, followed by four cycles of maintenance rituximab over the course of 1 year. Two months after initiation of chemotherapy (September 2014), she developed left lower extremity ataxia, which slowly progressed to involve her left arm followed by her right leg and right arm (by March of 2016). She also experienced severe vertigo exacerbated by any head movement but no diplopia or dysarthria.

Prior evaluation had included a brain magnetic resonance imaging (MRI) that showed scattered non-specific T2/FLAIR hyperintensities and atrophy of the cerebellar vermis and hemispheres. Routine cerebrospinal fluid (CSF) analyses including IgG index and oligoclonal bands were unremarkable. Serum paraneoplastic panel, copper/ceruloplasmin, vitamin levels (B1, B12, E), and autoimmune markers (i.e., celiac panel, antiGAD65 antibody) were negative.

She was admitted in June 2016 for an expedited evaluation after seeking out a second opinion. On admission she was wheelchair-bound, had severe appendicular and truncal ataxia, cerebellar dysarthria, and fatigable end-gaze direction-changing horizontal nystagmus. Cognition, strength, and sensation were intact. Biceps, brachioradialis, triceps, and patellar reflexes were 3/4 bilaterally and Achilles reflexes were 2/4 bilaterally. Plantar reflexes were flexor bilaterally.

**Fig. 1:**
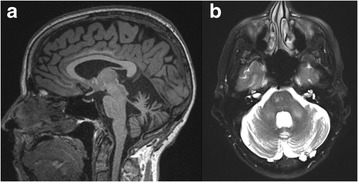
**a** Sagittal T1 MRI demonstrates severe atrophy of the cerebellar vermis and hemispheres. **b** Axial T2 MRI demonstrates patchy hyperintensities involving the pons and middle cerebellar peduncles and ex vacuo dilatation of the 4th ventricle

Serum immunoglobulins were within normal limits, CD20+ lymphocyte percentage was 5.9%, absolute CD4+ cell count was 212/mm^3^, and HIV was negative. Serum autoimmune labs including Thyroglobulin antibody (Ab), Thyroperoxidase Ab, Anti-Ri/La/Hu Abs, anti-VGCC Ab, anti-endomysial Ab, and Celiac panel were negative. In addition, the CSF Mayo encephalopathy panel (anti-NMDA receptor, anti-GABA, anti-ANNA-1, anti-ANNA-2, anti-GAD65 Abs) was negative. ANA was positive at a value of 1:80. Repeat brain MRI demonstrated worsening cerebellar atrophy and patchy T2/FLAIR hyperintensities involving the pons and middle cerebellar peduncles (Figure 1). FDG-PET/CT scan reported no abnormal FDG activity in the visualized brain. The scan showed asymmetric lymphoid activity in the retropharyngeal space (left > right), possibly physiologic or inflammatory. Flexible Fiberoptic Laryngoscopy to follow-up this finding was negative for any mucosal lesions or masses. Repeat CSF analysis revealed 2 WBCs/mm^3^, 148 RBCs/mm^3^, glucose 70 mg/dL, protein 37.7 mg/dL and negative flow cytometry/cytopathology. Notably, standard CSF JC virus PCR was negative. However, ultrasensitive TaqMan real-time JC virus PCR testing was positive at 15,180 copies/ml, consistent with a diagnosis of JC virus-related GCN.

Following her diagnostic admission in early July of 2016, she was enrolled in a clinical trial evaluating the efficacy and safety of pembrolizumab, a PD-1 receptor blocking antibody, in JC-virus associated disease. Quantitative JCV PCR load increased from 15,000 to 26,000 following the first round of treatment and her clinical status deteriorated further. Given the lack of clinical response and rising JC viral titer, treatment was discontinued and her cerebellar ataxia relentlessly progressed necessitating home hospice.

## Discussion

In one review examining PML after rituximab in HIV-negative patients (most with hematologic malignancies), the median time from first rituximab dose to onset of PML was 16 months (range of 1–90 months) [5]. GCN following treatment with rituximab has been described in only a single case report in which a patient was treated for Non-Hodgkin’s lymphoma with monthly rituximab for 11 years prior to developing symptoms [3]. In the case presented here, the patient had symptom onset only 2 months after initiation of rituximab and bendamustine, with continued progression while on chronic rituximab over the next 18 months.

PML was first discovered in three patients with underlying lymphoproliferative disorders, and in a case review of 230 cases of PML in the pre-AIDS era nearly two thirds had lymphoproliferative disorders with the majority being B-cell diseases [6]. There has been only one reported case of GCN in a patient with lymphoma, and this patient was also treated with rituximab as discussed above [3], emphasizing the novelty of our case. Given the short latency from time of rituximab treatment to the development of initial neurological symptoms, we must consider that underlying lymphoma could in fact be the causative factor for GCN development in this patient, though the definite symptoms began several months after chemotherapy initiation. It is also possible that the combination of pre-existing lymphoma and rituximab treatment is what ultimately led to the JC virus-related GCN. Although, given the continuation of chemotherapy treatment throughout the onset and progression of symptoms, causation is impossible to definitively determine.

No matter the primary underlying precipitant(s), GCN represents a diagnostic dilemma, as patients may present with nonspecific chronic cerebellar symptoms, normal serum and CSF studies, and normal or nonspecific brain MRI findings. Brain biopsy is the gold standard for diagnosis of GCN. Although JC virus CSF PCR has a sensitivity of approximately 74% and a specificity of 95.8% in diagnosing PML, the sensitivity of JC virus PCR in diagnosing GCN has not yet been well established [7]. Traditional/standard JC virus PCR testing used by many labs has a reported threshold of >50 DNA copies/mL which may not, as shown here, be sensitive enough to detect disease. Ultrasensitive PCR can detect a viral load of ≥10 DNA copies/mL and may be necessary to diagnose cases of GCN if clinical suspicion remains high.

The prognosis and progression of GCN has not been well elucidated, with some cases describing stabilization of disease progression following immune reconstitution [2] and others describing progression of disease leading to death [3]. To date, no protocol exists for risk stratifying patients for development of PML prior to initiating immune-suppressing therapies other than natalizumab in multiple sclerosis. Because of the diagnostic challenges in identifying GCN and the devastating nature of the disease, a high threshold of suspicion should be maintained both in patients with lymphoproliferative disorders and in patients on rituximab or other immune-suppressing agents, even in cases in which symptoms develop shortly after initiation of therapy.

## Conclusions

This report discusses a case of GCN with onset of symptoms just 2 months after initiation of rituximab/bendamustine treatment for follicular low-grade B cell lymphoma. GCN following treatment with rituximab for lymphoma has only been described in a single case report, in which a patient was treated with monthly rituximab for 11 years prior to developing symptoms. This patient’s diagnosis was delayed partly due to the traditional JC virus CSF PCR testing being negative, with definitive diagnosis coming only after a positive ultrasensitive TaqMan real-time JC virus PCR was obtained. This report elucidates the need for heightened clinical awareness of the challenges in diagnosing GCN as well as the need for a high threshold of suspicion in patients with an immune-suppressing condition or on immune-suppressing agents, even shortly after initiation.

## Abbreviations

CSF: Cerebrospinal fluid
GCN: Granule cell neuronopathy
MRI: Magnetic resonance imaging
PML: Progressive multifocal leukoencephalopathy
